# Correlation between *RASSF1A* Methylation in Cell-Free DNA and the Prognosis of Cancer Patients: A Systematic Review and Meta-Analysis

**DOI:** 10.1155/2022/3458420

**Published:** 2022-04-28

**Authors:** Shuchen Chen, He Duan, Dewei Zhang, Gongping Sun

**Affiliations:** ^1^Medical Oncology Department of Thoracic Cancer 1, Cancer Hospital of China Medical University, Liaoning Cancer Hospital & Institute, Shenyang, 110042 Liaoning Province, China; ^2^Department of the Third General Surgery, The Fourth Affiliated Hospital of China Medical University, Shenyang, 110032 Liaoning Province, China

## Abstract

**Background:**

Although the effects of methylation of the Ras association domain-containing protein 1 isoform A (*RASSF1A*) gene in cell-free DNA on the outcomes of patients with different types of cancer have been reported, the results are inconsistent.

**Objective:**

: To explore the relationships between *RASSF1A* methylation in cell-free DNA and the outcomes of cancer patients.

**Methods:**

The PubMed, Embase, and Web of Science databases were searched for papers related to this topic on December 8, 2021. The retrieved articles were screened by two independent researchers, following which the methodological quality of the selected studies was evaluated using the Newcastle-Ottawa Scale. Additionally, hazard ratios were calculated, and publication bias of the studies was determined using Egger's test.

**Results:**

Nine relevant publications involving a combined total of 1254 patients with different types of cancer were included in this study. The combined results of the random effects models yielded a hazard ratio of 1.73 (95% confidence interval: 1.31, 2.29; *P* < 0.001), which suggested there was a significant association between *RASSF1A* methylation and overall survival, and patients with an *RASSF1A* methylation status had a significantly increased risk of total death. Moreover, the Egger test result suggested there was no significant publication bias among the included studies.

**Conclusions:**

The methylation of *RASSF1A* in cell-free DNA in cancer patients was observably associated with an increased risk of poor overall survival.

## 1. Introduction

DNA methylation, a form of epigenetic modification, plays an important role in many physiological and pathophysiological processes, including carcinogenesis [1–3]. Epigenetic changes that may be tumor specific can be analyzed in circulating cell-free DNA (cfDNA) [4, 5]. To date, several types of cfDNA changes in cancer patients have been reported, including point mutations, loss of heterozygosity, and microsatellite instability [6]. Moreover, high concentrations of cfDNA circulating in the blood of cancer patients can be used as a marker to determine the origin of tumors. Although previous studies have demonstrated the predictive role of cfDNA methylation in cancer patients [7], the biological significance of cfDNAs remains largely unknown.

The Ras association domain-containing protein 1 isoform A (*RASSF1A*) gene, which encodes a member of the Ras association domain family, belongs to a class of genes that are often silenced by methylation rather than by mutation events. The RASSF1A protein is associated with the cell cycle, microtubule stabilization, and apoptosis [8, 9]. *RASSF1A* is usually expressed in most normal tissues, but it is downregulated or lost in some tumor cell lines and tissues [10]. The abnormal methylation of *RASSF1A* is associated with various types of tumors, including lung cancer and esophageal squamous carcinoma [11, 12]. Although several studies have reported the effect of *RASSF1A* methylation on the prognosis of patients with different types of cancer, the results are inconsistent across different studies [13–15] and vary even within the same cancer type, such as gastric cancer (GC) [13, 16] and breast cancer (BC) [14, 17]. Hence, to obtain more comprehensive and objective findings, we conducted a meta-analysis in this study to explore the correlation between *RASSF1A* methylation in cfDNA and the prognosis of cancer patients.

## 2. Methods

This meta-analysis was designed and analyzed in accordance with the guidelines of the Meta-analysis of Observational Studies in Epidemiology statement.

### 2.1. Search Strategy

A systematic search of the PubMed, Embase, and Web of Science databases [18] was conducted, using the keywords “cell-free circulating DNA,” “circulating tumor DNA,” “RASSF1A,” “methylation,” “methylated,” “neoplasm,” “cancer,” and “tumor.” Keywords in the same category were combined with “OR,” whereas those in different categories were combined with “AND.” Details of the complete search strategy used for each database are summarized in Supplementary Tables S1–S3. The search was last updated on December 8, 2021, without language restrictions. Hence, to obtain more studies that could be used for the meta-analysis, a manual retrieval of literature articles was also conducted, and relevant references in reviews and included research papers were screened.

### 2.2. Study Selection

After the literature search, the retrieved articles were further screened according to certain inclusion and exclusion criteria. The inclusion criteria were as follows: (1) the study participants were adult patients with a histologically or pathologically confirmed cancer; (2) the study investigated the relationship between *RASSF1A* methylation in blood cfDNA and patient outcomes; (3) the study was of cohort type; and (4) the study reported hazard ratios (HRs) with 95% confidence intervals (95% CIs) of overall survival (OS), progression-free survival (PFS), disease-free survival (DFS), or relapse-free survival (RFS), adjusted by univariate or multivariable analysis.

The exclusion criteria were as follows: (1) reviews, conference abstracts, commentaries, and other nonliterary research articles; (2) studies in which HRs (95% CIs) were not reported or could not be calculated according to the information in the article; and (3) repeated publications or multiple articles with the same data (only the one with the most complete research information was included).

### 2.3. Data Extraction and Quality Assessment

The data extraction according to the inclusion and exclusion criteria described above was performed by two reviewers. The following data were extracted: the first author name, year of publication, country in which the research was conducted, research type, basic characteristics of the object of study (sample size), patient gender, patient age, cancer type, sampling time, follow-up time, confounding factors, and research outcomes. After completing the data extraction, the two reviewers would exchange their extraction tables and discuss and resolve any inconsistencies.

The methodological quality of the studies, which was evaluated on the basis of the selection, comparability, and exposure of the included subjects, was scored according to the Newcastle-Ottawa Scale (NOS; 8 scoring items, full score of 9 points) [19]. Studies with a score of 7–9 were considered of high-quality research, those with a score of 4–6 were considered of medium quality, and those with a score of less than 4 were considered of low quality.

### 2.4. Statistical Analysis

To explore the association between the *RASSF1A* methylation status and the prognosis of cancer patients, HRs and 95% CIs were used as effect size indicators for evaluating whether the difference in risk of death between the methylated and unmethylated groups was statistically significant. Cochran's *Q* test and the *I*^2^ test were used to test for heterogeneity [20]. A *Q* statistic of *P* < 0.05 or *I*^2^ > 50% indicates that there is significant heterogeneity between studies, and the random effects model should be used for the meta-analysis. Values of *P* ≥ 0.05 and *I*^2^ ≤ 50% suggest that heterogeneity is not significant, and the fixed effects model is used instead. In addition to the inclusion of all cancer patients for the meta-analysis, the effect of *RASSF1A* methylation on patient prognosis was also evaluated according to specific cancer types. Egger's test was used to assess whether there was significant publication bias in the included studies [21]. Additionally, the stability of the results was tested using the one-by-one elimination method [22].

## 3. Results

### 3.1. Study Selection

**Figure 1:**
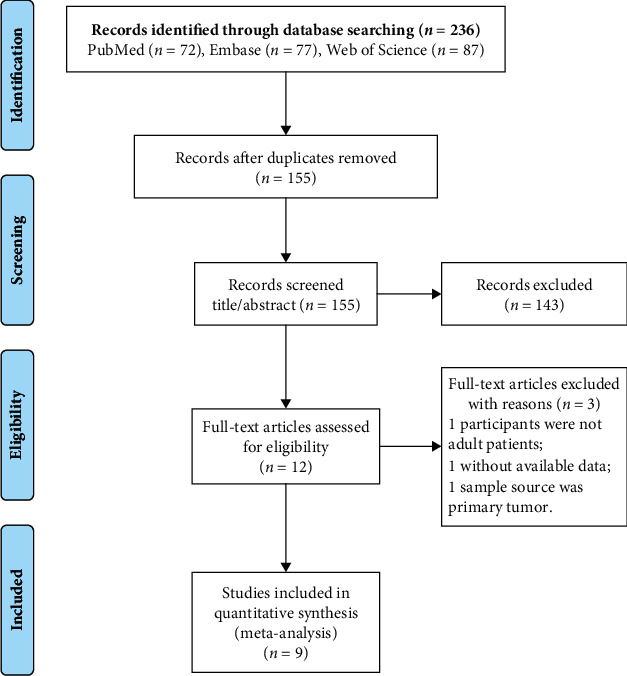
Flow diagram of the literature search process for selecting studies for the meta-analysis.

Figure 1 illustrates the article retrieval process, where a total of 236 citations (72 from PubMed, 77 from Embase, and 87 from Web of Science) were initially identified. After removing 81 duplicate articles, 155 were retained for evaluation of their titles and abstracts, whereupon 143 articles that did not meet the inclusion criteria were removed. Of the 12 remaining articles, one study with children as the research object, one study with tumors as the sample, and one study with no available data were excluded after reading of the full texts. Finally, the nine remaining articles [13–17, 23–26] were selected for the meta-analysis, as the manual retrieval failed to find any additional reports that could be included.

### 3.2. Research Characteristics and Quality Evaluation

As indicated in Table 1, the nine articles included in this study spanned the publication years of 2005–2021 and were conducted in Greece, Austria, Thailand, Denmark, and Iran. The sample size ranged from 61 to 357, with a combined total of 1254 patients (469 males and 785 females), whose malignancies included GC, BC, colorectal cancer (CRC), and prostate cancer. Because the study by Matthaios et al. [15] reported data for both early and metastatic CRC, there were 10 sets of data in our meta-analysis. Except for the study by Gӧbel et al. [17], all the other studies had sampled their patients before treatment. All nine studies used the methylation-specific polymerase chain reaction to detect the *RASSF1A* methylation status in cfDNA. Only three studies reported an HR (95% CI) with multifactor adjustment, whereas the remaining studies conducted single-factor analyses.

The results of the methodological quality evaluations are shown in Table 2. The NOS scores of the included studies ranged from 6 to 8. Of the nine studies, four were of medium methodological quality and five were of high quality. The main bias types were recall bias and confounding bias.

### 3.3. Results of the Meta-Analysis

**Figure 2:**
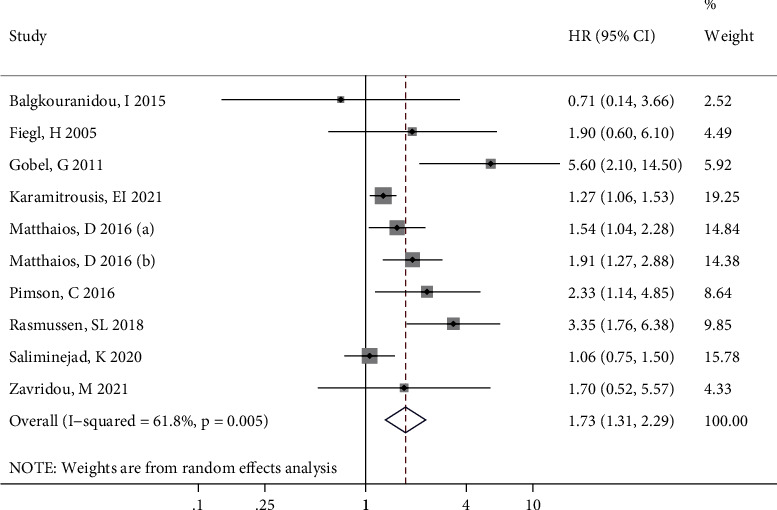
Forest plot showing the association of *RASSF1A* methylation with overall survival in all cancers.

Since only one article reported RFS, DFS, and PFS results, this meta-analysis only incorporated effect values on OS.  Figure 2 shows the forest plot of the associations between *RASSF1A* methylation and OS for all the cancer patients, where it can be seen that there was significant statistical heterogeneity among the included studies. The combined results of the random effect models suggested a significant relationship between *RASSF1A* methylation and OS (*P* < 0.001). Therefore, cancer patients with *RASSF1A* methylated may have a significantly increased risk of a poor survival prognosis.

**Figure 3:**
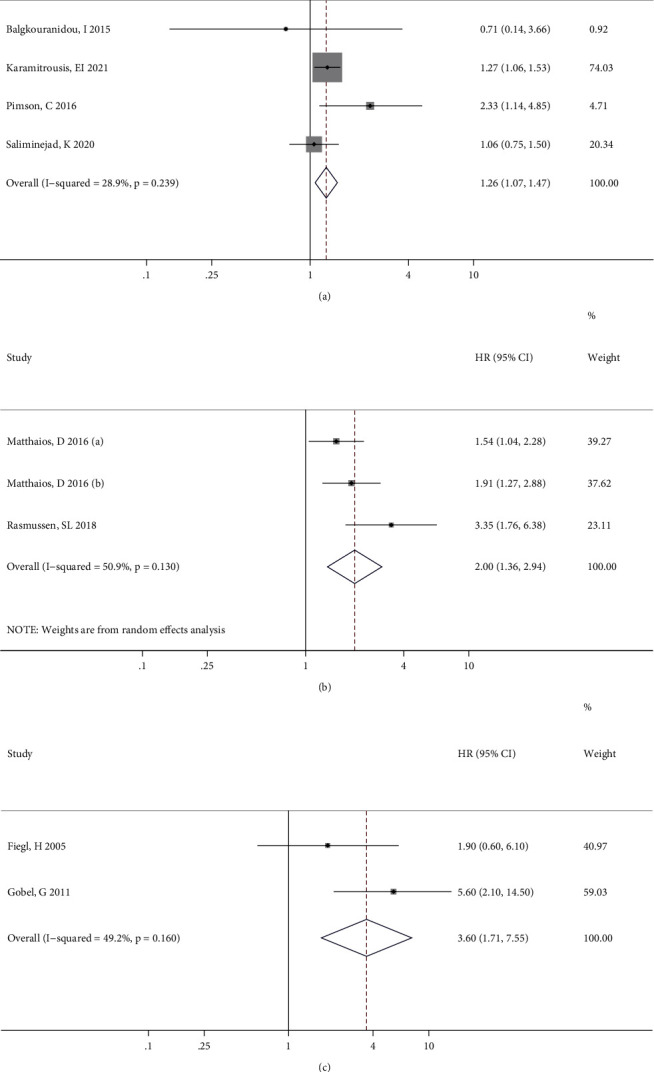
Forest plots showing the associations of *RASSF1A* methylation with overall survival in patients with gastric cancer (a), colorectal cancer (b), and breast cancer (c).

The meta-analysis results of the association between *RASSF1A* methylation and OS in patients with GC, CRC, and BC specifically are illustrated in Figures 3(a)–3(c), respectively. For GC patients, there was no significant heterogeneity between studies, and the combined HR (95% CI) in the fixed effects model indicated a significant positive correlation (*P* = 0.004) between *RASSF1A* methylation and GC prognosis (Figure 3(a)). By contrast, although the effect values for the CRC patients showed significant interstudy heterogeneity, the pooled results of the random effects model suggested a close relationship (*P* < 0.001) between *RASSF1A* methylation and CRC prognosis (Figure 3(b)). Furthermore, only two studies reported outcomes in BC patients, with no significant interstudy heterogeneity, and the pooled HR (95% CI) in the fixed effects model indicated a significant increased risk of a poor prognosis for patients with *RASSF1A* methylated in their cfDNA (*P* = 0.022; Figure 3(c)).

### 3.4. Sensitivity Analysis

As shown in Table 3, for all the included studies, the sensitivity analysis suggested that the combined HR (95% CI) remained robust after removing any one study (*P* < 0.05), and the combined results were stable and not affected by any single study. Similarly, the sensitivity analysis of the CRC patients also suggested stable combined outcomes. However, the meta-analysis results for the GC patients were not stable because they became insignificant (*P* = 0.238) after the Karamitrousis [16] study had been excluded.

### 3.5. Risk of Publication Bias

Egger's test, which was used to evaluate whether there was observable publication bias between the studies, yielded *P* values of greater than 0.05 for the total, GC, and CRC studies, indicating the absence of any publication bias (Table 3). The limited numbers of BC and castration-resistant prostate cancer studies (*n* = 2 and 1, respectively) rendered them unsuitable for sensitivity analysis or Egger's test.

## 4. Discussion

*RASSF1A*, a well-known tumor suppressor gene, affects cancer development and progression. Its inactivation through mutation, loss of heterozygosity, or promoter methylation occurs in various cancer types. The effect of *RASSF1A* methylation on the prognosis of patients with specific types of cancer has been well reported, whereas there are no published studies on the association between *RASSF1A* methylation in cfDNA and prognosis for all cancer patients. Therefore, we conducted this meta-analysis and found significant correlations between *RASSF1A* methylation in cfDNA and cancer prognosis in general, especially in GC, CRC, and BC.

*RASSF1A* methylation is considered to be one of the first cellular changes to occur during tumor progression, and the frequency of this epigenetic modification varies widely among solid tumors [27, 28]. The prognostic role of such abnormal methylation of *RASSF1A* has also been investigated. Hassan et al. found that methylation of *RASSF1A* was associated with poor prognosis in patients with neuroblastoma [29]. Sensitivity analysis of another meta-analysis showed that *RASSF1A* methylation in lung cancer tissues was significantly associated with a lower OS [30]. These findings support our conclusions to a certain extent. Furthermore, the inactivation of *RASSF1A* is often caused by hypermethylation of the promoter region, which allows the abnormal methylation of *RASSF1A* being more easily detected in a variety of cancers, including BC, lung cancer, and gastrointestinal cancer [31]. The study has further confirmed that cases of hepatocellular carcinoma with *RASSF1A* promoter hypermethylation had poor prognoses [32]. Compared with patients without methylation, GC patients with *RASSF1A* promoter methylation had significant lower PSF and OS [16]. Although *RASSF1A* remains expressed in cancer, concentrations of RASSF1A protein above or below its optimal expression threshold can disrupt signal transduction and lead to malignant transformation [31]. Promoter methylation of *RASSF1A* was also found to be significantly correlated with tumor size and histological grade of BC [33]. Therefore, we hypothesized that hypermethylation of *RASSF1A* promoter disrupts normal body signal transduction, leading to tumor recurrence, and metastasis and poorer clinical outcomes.

Clinically, liquid biopsy or collection and analysis of easily accessible patient samples, such as blood, can contribute to the diagnosis of early tumors, as a less invasive diagnostic approach [34, 35]. CfDNAs are small fragments of DNA released from normal or tumor tissues as a result of cell apoptosis or necrosis and can be captured in large quantities in the blood [36]. It has been suggested that the concentration of cfDNA in the blood of cancer patients tends to be higher, because increased tumor size will promote cell apoptosis and necrosis, thus releasing a large amount of cfDNAs [37]. Furthermore, epigenetic biomarkers are stable and highly tissue specific in body fluids [38]. Therefore, this study proposed a potential biomarker, methylation of *RASSF1A* in cfDNA, that can easily be detected in liquid biopsy samples or blood samples from cancer patients to predict their prognostic risks and clinical outcomes.

To illustrate the strengths of this meta-analysis, we not only included patients with various types of cancer for the combination of effect values but also grouped specific cancers for analysis, with the combination results showing good consistency. Furthermore, the evaluation of the methodological quality of the included studies showed that selection bias, measurement bias, and lost track bias were reasonably controlled (although recall bias and confounding bias existed to a certain extent), which suggested the studies were of medium and high quality. More importantly, no significant publication bias was found in this meta-analysis, and the combined results were highly reliable.

There were several limitations to our meta-analysis. First, although the statistical heterogeneity of the nine studies was relatively small, the clinical and methodological heterogeneity among them (e.g., the impacts of cancer stage and treatment on patient prognosis) should not be ignored. Second, the number of studies included in the meta-analysis was small, with less than five articles for each specific cancer type. Additionally, the incomplete literature information made it difficult for us to explore the influences of age, cancer stage, treatment methods, and other factors on the combined results through subgroup analysis. Third, because the meta-analysis results for GC and BC were unstable, more large-sample studies are needed to verify the findings. Finally, it is recommended that follow-up studies focus on the relationships between *RASSF1A* methylation and disease progression and recurrence for comparison with the OS association.

In conclusion, this meta-analysis revealed that *RASSF1A* methylation in cfDNA was observably associated with an increased risk of total deaths for patients with GC, CRC, and BC. Therefore, more high-quality, large-sample, and prospective cohort studies are recommended to verify the stability of the results.

## Figures and Tables

**Table 1:** Details of the nine included studies.

Study	Country	Study type	Patients	Timing sample	*n*, M/F	Age, years	Methylation, Y/N	Follow-up, months	Outcomes
Balgkouranidouet al. [13]	Greece	NR	GC	Preoperatively	73, 51/22	67.07 ± 11.09	50/23	56 (12–111)^$^	OS
Fiegl et al. [14]	Austria	PCS	BC	Pretherapeutic	148, 0/148	62 (37–88)^$^	29/119	48 (6–117)^$^	OS, RFS
Gӧbel et al. [17]	Austria	PCS	BC	During surgery	357, 0/357	57.4 (50.0, 66.3)^#^	78/279	51 (35, 68)^#^	OS, DFS
Karamitrousis et al. [16]	Greece	NR	GC	Pre-therapeutic	70, 43/27	19 ≤ 60/51 > 60	52/18	Up to 48	OS, PFS
Matthaios et al. [15]	Greece	NR	eCRC	Preoperatively	88, 51/37	70 (44–76)^$^	22/66	Up to 111	OS
			mCRC	Pretherapeutic	67, 38/29	70 (44–76)^$^	30/37	Up to 67	OS
Pimson et al. [23]	Thailand	NR	GC	Pretherapeutic	101, 44/57	17 ≤ 40/25 41–50/29 51–60/30 > 67	84/17	Up to 120	OS
Rasmussen et al. [24]	Denmark	RCS	CRC	Pretherapeutic	193, 119/74	91 ≤ 67/102 > 67	22/171	Up to 60	OS
Saliminejad et al. [25]	Iran	NR	GC	Pretherapeutic	96, 62/34	59.5 ± 12.3	32/64	Median 20	OS
Zavridou et al. [26]	Greece	PCS	mCRPC	Pretherapeutic	61, 61/0	NR	14/47	Up to 60	OS

PCS: prospective cohort study; RCS: retrospective cohort study; NR: not reported; BC: breast cancer; CRC: colorectal cancer; eCRC: early operable colorectal cancer; mCRC: metastatic colorectal cancer; mCRPC: metastatic castration-resistant prostate cancer; GC: gastric cancer; M: male; F: female; Y: yes; N: no; OS: overall survival; RFS: relapse-free survival; DFS: disease-free survival; PFS: progression-free survival. ^$^Median (range); ^#^Median (interquartile range).

**Table 2:** Quality assessment of the included studies.

Study	Representativeness of the exposed cohort	Selection of the unexposed cohort	Ascertainment of exposure	Outcome of interest not present at start of study	Control for important factor or additional factor	Outcome assessment	Follow-up long enough for outcomes to occur	Adequacy of follow-up of cohorts	Total quality scores
Balgkouranidou et al. [13]	☆	☆	☆	—	—	☆	☆	☆	6
Fiegl et al. [14]	☆	☆	☆	☆	—	☆	☆	☆	7
Gӧbel, et al. [17]	☆	☆	☆	☆	☆	☆	☆	☆	8
Karamitrousis et al. [16]	☆	☆	☆	—	—	☆	☆	☆	6
Matthaios et al. [15]	☆	☆	☆	—	—	☆	☆	☆	6
Pimson et al. [23]	☆	☆	☆	—	☆	☆	☆	☆	7
Rasmussen et al. [24]	☆	☆	☆	—	☆☆	☆	☆	☆	8
Saliminejad et al. [25]	☆	☆	☆	—	—	☆	☆	☆	6
Zavridou et al. [26]	☆	☆	☆	☆	—	☆	☆	☆	7

**Table 3:** Outcomes of the sensitivity analysis and publication bias test.

Patients	No. of studies	Sensitivity analysis		Egger' s test
		HR (95% CI)	Robust	*P* value
Total	10	1.58 (1.22, 2.04) to 1.91 (1.40, 2.60)	Yes	0.097
GC	4	1.20 (0.88, 1.64) to 1.31 (1.10, 1.56)	No	0.922
CRC	3	1.71 (1.29, 2.27) to 2.39 (1.39, 4.10)	Yes	0.172

GC: gastric cancer; CRC: colorectal cancer; HR: hazard ratio; 95% CI: 95% confidence interval.

## Data Availability

The data availability declaration is applicable.

## Supplementary Materials

Supplementary Materials Supplementary Table S1: PubMed search strategy. Supplementary Table S2: Embase search strategy. Supplementary Table S3: Web of Science search strategy.
