# Supplementary material for: Correlation between RASSF1A Methylation in Cell-Free DNA and the Prognosis of Cancer Patients: A Systematic Review and Meta-Analysis
Source: J Oncol. 2022 Apr 28;2022:3458420. doi: 10.1155/2022/3458420 (PMC9071870; doi:10.1155/2022/3458420)
Supplement: Supplementary Materials — Supplementary Table S1: PubMed search strategy. Supplementary Table S2: Embase search strategy. Supplementary Table S3: Web of Science search strategy. [file 3458420.f1.docx]

**Sup Table 1. Search strategy of PubMed**

| Search | Query | Items found |
| --- | --- | --- |
| #1 | ("circulating tumor dna"[MeSH Terms] OR ("circulating"[All Fields] AND "tumor"[All Fields] AND "dna"[All Fields]) OR "circulating tumor dna"[All Fields] OR "ctdna"[All Fields] OR "ctdnas"[All Fields]) OR ("cell free nucleic acids"[MeSH Terms] OR ("cell free"[All Fields] AND "nucleic"[All Fields] AND "acids"[All Fields]) OR "cell free nucleic acids"[All Fields] OR "cell free circulating dna"[All Fields] OR "cfdna"[All Fields] OR "cfdnas"[All Fields]) | 14240 |
| #2 | "RASSF1A"[All Fields] | 1525 |
| #3 | "methylation"[MeSH Terms] OR "methyl"[All Fields] OR "methylate"[All Fields] OR "methylated"[All Fields] OR "methylates"[All Fields] OR "methylating"[All Fields] OR "methylation"[All Fields] OR "methylations"[All Fields] OR "methylational"[All Fields] OR "methylator"[All Fields] OR "methylators"[All Fields] OR "methyls"[All Fields] | 471491 |
| #4 | "neoplasms"[MeSH Terms] OR "neoplasms"[All Fields] OR "neoplasm"[All Fields] OR "cancerated"[All Fields] OR "canceration"[All Fields] OR "cancerization"[All Fields] OR "cancerized"[All Fields] OR "cancerous"[All Fields] OR "cancer"[All Fields] OR "cancers"[All Fields] OR "cysts"[MeSH Terms] OR "cysts"[All Fields] OR "cyst"[All Fields] OR "neurofibroma"[MeSH Terms] OR "neurofibroma"[All Fields] OR "neurofibromas"[All Fields] OR "tumoral"[All Fields] OR "tumorous"[All Fields] OR "tumour"[All Fields] OR "tumor"[All Fields] OR "tumoural"[All Fields] OR "tumourous"[All Fields] OR "tumours"[All Fields] OR "tumors"[All Fields] | 5058158 |
| #5 | #1 AND #2 AND #3 AND #4 | 72 |

**Sup Table 2. Search strategy of Embase**

| Search | Query | Items found |
| --- | --- | --- |
| #1 | ('ctdna'/exp OR ctdna OR cfdna OR 'circulating tumor dna'/exp OR 'circulating tumor dna' OR 'cell free circulating dna'/exp OR 'cell free circulating dna' OR 'cell free dna'/exp OR 'cell free dna') | 18935 |
| #2 | ('neoplasm'/exp OR neoplasm OR 'cancer'/exp OR cancer OR 'tumor'/exp OR tumor OR 'tumour'/exp OR tumour) | 5757265 |
| #3 | ('methyl'/exp OR 'methyl' OR 'methylate' OR 'methylated' OR 'methylates' OR 'methylating' OR 'methylation'/exp OR 'methylation' OR 'methylations' OR 'methylational' OR 'methylator' OR 'methylators' OR 'methyls') | 617322 |
| #4 | rassf1a | 1909 |
| #5 | #1 AND #2 AND #3 AND #4 | 77 |

**Sup Table 3. Search strategy of Web of Science**

| Search | Query | Items found |
| --- | --- | --- |
| #1 | ctDNA OR cfDNA OR (circulating tumor dna) OR (cell free circulating dna) (All Fields) | 14550 |
| #2 | neoplasm OR cancer OR tumor OR tumour (All Fields) | 4755634 |
| #3 | "methylation" OR "methyl" OR "methylate" OR "methylated" OR "methylates" OR "methylating" OR "methylation" OR "methylations" OR "methylational" OR "methylator" OR "methylators" OR "methyls" (All Fields) | 719239 |
| #4 | RASSF1A (All Fields) | 1857 |
| #5 | #1 AND #2 AND #3 AND #4 | 87 |
